# Mapping visuospatial attention: the greyscales task in combination with repetitive navigated transcranial magnetic stimulation

**DOI:** 10.1186/s12868-018-0440-1

**Published:** 2018-07-11

**Authors:** Katrin Giglhuber, Stefanie Maurer, Claus Zimmer, Bernhard Meyer, Sandro M. Krieg

**Affiliations:** 1 Department of Neurosurgery, Klinikum rechts der Isar, Technische Universität München, Ismaninger Str. 22, 81675 Munich, Germany; 2 TUM-Neuroimaging Center, Klinikum rechts der Isar, Technische Universität München, Munich, Germany; 3 Section of Neuroradiology, Department of Radiology, Klinikum rechts der Isar, Technische Universität München, Ismaninger Str. 22, 81675 Munich, Germany

**Keywords:** Cortical mapping, Greyscales task, Neglect, Repetitive navigated transcranial magnetic stimulation, Tachistoscopic testing, Visuospatial attention

## Abstract

**Background:**

Visuospatial attention is executed by the frontoparietal cortical areas of the brain. Damage to these areas can result in visual neglect. We therefore aimed to assess a combination of the greyscales task and repetitive navigated transcranial magnetic stimulation (rTMS) to identify cortical regions involved in visuospatial attention processes. This pilot study was designed to evaluate an approach in a cohort of healthy volunteers, with the future aim of using this technique to map brain tumor patients before surgery. Ten healthy, right-handed subjects underwent rTMS mapping of 52 cortical spots in both hemispheres. The greyscales task was presented tachistoscopically and was time-locked to rTMS pulses. The task pictures showed pairs of horizontal rectangles shaded continuously from black at one end to white at the other, mirror-reversed. On each picture the subject was asked to report which of the two greyscales appeared darker overall. The responses were categorized into “leftward” and “rightward,” depending on whether the subject had chosen the rectangle with the darker end on the left or the right. rTMS applied to cortical areas involved in visuospatial attention is supposed to affect lateral shifts in spatial bias. These shifts result in an altered performance on the greyscales task compared to the baseline performance without rTMS stimulation.

**Results:**

In baseline conditions, 9/10 subjects showed classic pseudoneglect to the left. Leftward effects also occurred more often in mapping conditions. Yet, calculated rightward deviations were strikingly greater in magnitude (*p* < 0.0001). Overall, the right hemisphere was found to be more suggestible than the left hemisphere. Both rightward and leftward deviation scores were higher for the rTMS of this brain side (*p* < 0.0001). Right hemispheric distributions accord well with current models of visuospatial attention (Corbetta et al. Nat Neurosci 8(11):1603–1610, 2005). We observed leftward deviations triggered by rTMS within superior frontal and posterior parietal areas and rightward deviations within inferior frontal areas and the temporoparietal junction (TPJ).

**Conclusion:**

The greyscales task, in combination with rTMS, yields encouraging results in the examination of the visuospatial attention function. Future clinical implications should be evaluated.

**Electronic supplementary material:**

The online version of this article (10.1186/s12868-018-0440-1) contains supplementary material, which is available to authorized users.

## Background

Visuospatial attention is processed in particular brain areas and fiber tract connections [2, 3]. The complexity of interactions becomes apparent by regarding the corresponding pathology at malfunction: visual neglect. Visual neglect describes a neurological syndrome of various forms, degrees, and recovery potential, accompanied by a significantly reduced functional outcome [4–6]. Classically observed as a consequence of right hemispheric parietal lesions, it has also been reported after left hemispheric, frontal, temporal, subcortical, and combined brain lesions [7, 8]. Research on detecting and understanding the underlying mechanisms is essential. In tumor patients, mapping prior to resection may prevent functional deficits [9, 10]. In stroke patients, mapping and timely counteraction may prevent chronification [1, 11–13].

To learn more about the visuospatial attention function, it proved insightful to study the conditions of healthy adults. As frequently reported, and also meta-analyzed by Jewell and McCourt in 2000, neurologically healthy individuals show slight but significant leftward errors in line bisection tasks [14–19]. Bowers and Heilman described this phenomenon first, calling it “pseudoneglect” [20]. Common models ascribe this observation to a right-hemispheric dominance in spatial attention processing. Imaging studies show preferential activity of the right hemisphere during visuospatial task performance [16, 21]. Other projects have examined the effect of inactivating the right hemisphere and have reported both activity shifts to the left hemisphere and a resultantly reduced leftward bias [14, 22, 23]. In 2011, Thiebaut de Schotten et al. confirmed anatomical correlates. They were able to link pseudoneglect to a larger network of frontoparietal fiber tracts within the right hemisphere compared to the left hemisphere [24]. Conclusively, Varnava et al. studied the predictability of visuospatial deficits depending on the extent and direction of pseudoneglect in the initial state. Reasoning from their findings, pseudoneglect and neglect originate from common or at least coupled mechanisms [25].

Conventional neglect screening in patients is usually undertaken using paper-and-pencil tests (e.g., line bisection). However, to measure biases in perceptual attention sensitively, task and setting must be selected appropriately [26]. As for measuring pseudoneglect in healthy volunteers, the greyscales task by Mattingley et al. consistently obtained promising results. First describing the test in 1994, they proved its sensitivity in several studies and developed an electronic version [27–30]. The task consists of tachistoscopic forced-choice decisions on the luminance of two greyscales. Analysis results in a score reflecting the spatial bias. The score ranges from − 1.00, reflecting a maximal leftward bias, to 1.00, for the right side, respectively.

Repetitive navigated transcranial magnetic stimulation (rTMS) affords an opportunity to accurately and non-invasively detect cortical areas. rTMS pulses applied to an eloquent cortical spot effect a so-called virtual lesion and thus temporary inactivation. As a result, we can observe performance changes on concurrently conducted neuropsychological tasks. The method is increasingly used to map neuropsychological functions such as language and calculation; recently, our group also reported its usefulness for the mapping of visuospatial attention [31–37]. To further pursue this objective, we combined rTMS with the aforementioned greyscales task in the same cohort of healthy volunteers as investigated before [36]. We assumed our subjects present with a basic spatial bias that reflects their individual processing balance between the left and the right hemisphere. This bias might be indexed by the greyscales task. In the presence of pseudoneglect, we would obtain leftward baseline scores. Our next thought was that temporary inactivation of eloquent cortical spots ought to effect an inter-hemispheric misbalancing and therefore drive lateral shifts in spatial bias. These again might be indexed by the greyscales task. We expected particularly significant effects for rTMS applied to cortical spots of the right hemisphere. Based on the idea of a dominantly active right hemisphere in healthy adults with pseudoneglect, we supposed that inactivation of spots within this hemisphere would reduce the basic leftward bias. Hence we would obtain rightward deviation scores on the greyscales task.

Summarizing, the presented pilot study aims to assess a combination of the greyscales task and rTMS in healthy volunteers by examining the following hypotheses:

1. The greyscales task in tachistoscopic test conditions is appropriate and sensitive for testing visuospatial attention function via rTMS.
2. The resulting brain maps are in accordance with current models of visuospatial attention.

## Methods

### Subjects

The study included five women and five men. All subjects were healthy at state and without any history of neurological or neuropsychological deficit. Their ages ranged from 21 to 31 years (median age: 24 years). Inclusion criteria were pure right-handedness (Edinburgh inventory score > 40) and German as a first language. Exclusion criteria were general TMS and MRI exclusion criteria (pacemaker, cochlea-implant, deep brain stimulation) [38]. As mentioned in the introduction, this cohort has been examined before [36].

### Navigated rTMS

#### MRI dataset

For MR imaging, we used a 3 Tesla MRI scanner with eight-channel phased-array head coil (Achieva 3T, Philips Medical Systems, Amsterdam, The Netherlands B.V.). Our protocol was comprised of two sequences: a T2-weighted FLAIR sequence (TR: 12,000 ms, TE: 140 ms, voxel size: 0.9 × 0.9 × 4 mm^3^, acquisition time: 3 min) and a T1-weighted 3D gradient echo sequence (no intravenous contrast administration, TR: 9 ms, TE: 4 ms, 1 mm^3^ isovoxel covering the whole head, acquisition time: 6 min 58 s). The 3D dataset was transferred to our rTMS system by DICOM standard.

#### Mapping setup

For rTMS mapping, we used a Nexstim eXimia System Version 4.3 with NEXSPEECH^®^ module (Nexstim Plc., Helsinki, Finland). This system uses a stereotactic camera to link the subject’s 3-D MRI dataset with its head via anatomical landmarks and a registered “tracker” headband. This meant we were able to visualize the stimulation coil’s real-time position or, rather, the induced electric field in the 3D MRI reconstruction and to selectively and accurately stimulate the brain regions [33, 34, 39, 40]. Through the use of NEXSPEECH software, we were able to stimulate the selected brain regions and time-locked present task pictures on a video screen [41].

#### Mapping parameter

In each subject we determined resting motor thresholds (RMT) for the right and left abductor pollicis brevis muscles and individually adjusted the stimulation intensity for the respectively contralateral hemisphere [42]. Mapping was performed at 100% RMT. rTMS pulses were applied as a train of 10 stimuli at a repetition frequency of 5 Hz, equaling stimulation trains of 1800 ms. To reach a maximal field induction, we placed the coil in anterior–posterior field orientation strictly tangentially to the skull, as previously reported [36, 41, 43].

#### Mapping targets

**Fig. 1:**
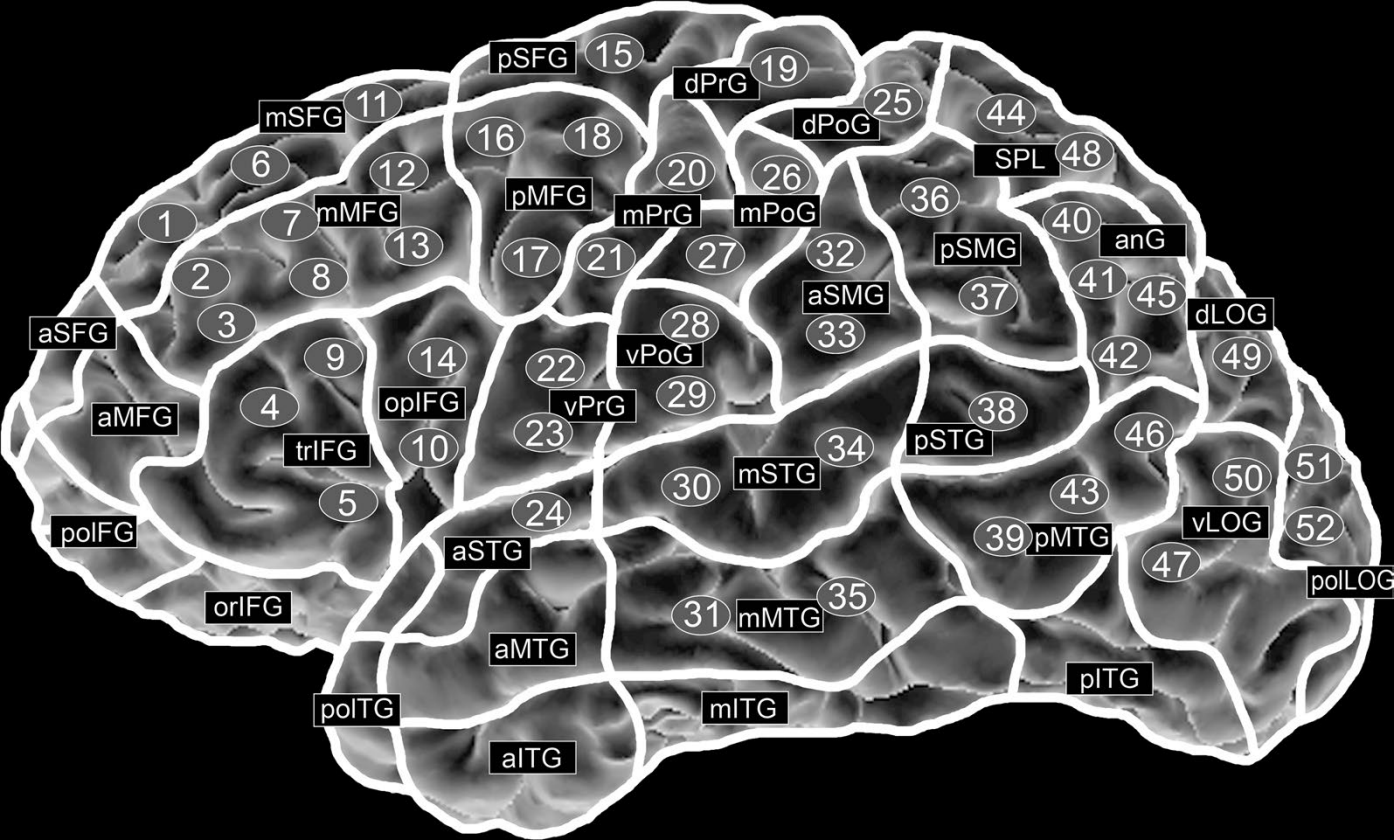
Mapping targets. Brain areas and cortical spots no. 1–52 according to the cortical parcellation system [44]

We tested 52 cortical spots on each hemisphere and distributed them to brain areas using the cortical parcellation system created by Corina (CPS; Fig. 1, Table 1) [44]. We anatomically identified the spots in each subject’s 3D MRI reconstruction and marked them as stimulation targets. First we selected the targets of the left hemisphere. We probed each target five times in a block. The order of selecting was randomly chosen by the examiner. Next we examined the right hemisphere, respectively. We redid this procedure once. According to this protocol each target was probed 10 times in total. Though, due to difficulties in adjusting the stereotactic camera during the mapping, some spots got addressed more, some less frequent. Certain brain areas had to be omitted: Stimulation of the polar and anterior frontal gyri (polFG, aSFG, aMFG), the orbital part of the inferior frontal gyrus (orIFG), the polar temporal gyri (polTG), and the anterior middle temporal gyrus (aMTG) is known to be too painful to provide reliable results due to muscle contractions. Stimulation of the inferior temporal gyrus (ITG) is known to be incomparably effective because the increased range between the skull and brain tissue causes decreased stimulation intensities [39, 45].

**Table 1:** Anatomical names and abbreviations of the cortical parcellation system

Abbreviation	Anatomy
aITG	Anterior inferior temporal gyrus
aMFG	Anterior middle frontal gyrus
aMTG	Anterior middle temporal gyrus
anG	Angular gyrus
aSFG	Anterior superior frontal gyrus
aSMG	Anterior supramarginal gyrus
aSTG	Anterior superior temporal gyrus
dLOG	Dorsal lateral occipital gyrus
dPoG	Dorsal post-central gyrus
dPrG	Dorsal pre-central gyrus
mITG	Middle inferior temporal gyrus
mMFG	Middle middle frontal gyrus
mMTG	Middle middle temporal gyrus
mPoG	Middle post-central gyrus
mPrG	Middle pre-central gyrus
mSFG	Middle superior frontal gyrus
mSTG	Middle superior temporal gyrus
opIFG	Opercular inferior frontal gyrus
orIFG	Orbital part of the inferior frontal gyrus
pITG	Posterior inferior temporal gyrus
pMFG	Posterior middle frontal gyrus
pMTG	Posterior middle temporal gyrus
polFG	Polar frontal gyri
polTG	Polar temporal gyri
polLOG	Polar lateral occipital gyrus
pSFG	Posterior superior frontal gyrus
pSMG	Posterior supramarginal gyrus
pSTG	Posterior superior temporal gyrus
SPL	Superior parietal lobe
trIFG	Triangular inferior frontal gyrus
vLOG	Ventral lateral occipital gyrus
vPoG	Ventral post-central gyrus
vPrG	Ventral pre-central gyrus

Anatomical names and abbreviations according to the cortical parcellation system [44]

### The greyscales task

#### Task setup

During rTMS mapping, the subjects had to perform a visuospatial attention task. More specifically, they had to handle one task picture during each rTMS stimulation train. A video screen (38.1 cm in diameter) was placed at viewing distance (about 60 cm nose to screen) in front of the examination chair. As evaluated before, we delivered rTMS pulses and task pictures synchronously and without delay between rTMS-stimulus-onset and picture-display [46]. The inter-picture interval was set to 3000 ms.

#### Task design

**Fig. 2:**
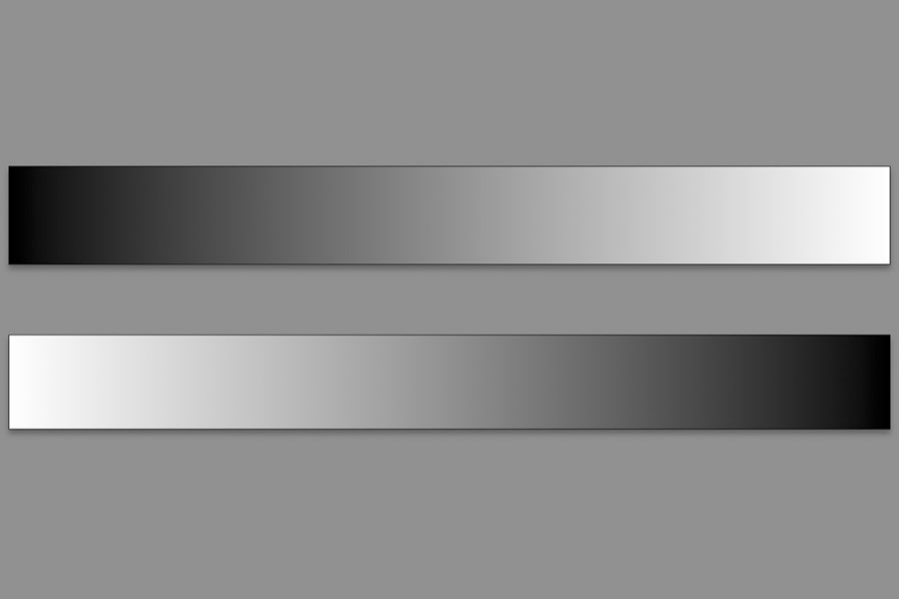
Sample picture from the greyscales task. Greyscales task sample. For each picture the subject was asked to report which of the two greyscales appeared to be darker overall. The responses were categorized into “leftward” and “rightward,” depending on whether the subject had chosen the rectangle with the darker end on the left or the right, as first described by Mattingley et al. [30]

Our visuospatial attention task follows the greyscales task by Mattingley et al. [30]. Task pictures were conceived as pairs of horizontal rectangles arranged vertically, one above another (Fig. 2). They were shaded continuously from black at one end to white at the other, shown on a grey background, and framed by a black line of 0.7 mm. The rectangles of each pair were identical in length and shading, solely depicted as mirror images. Uniformly 30 mm in height, the rectangles varied in length from 180 to 330 mm (in 30 mm steps). Six lengths per two shading orientations each made a task set of 12 different task pictures. Pictures were displayed tachistoscopically for 50 ms, as reported earlier [18, 36, 47]. The order of presentation was randomized by the software. On each picture the subject was asked to report which of the two greyscales appeared darker overall by saying aloud “top” or “bottom.” There was no third option to select “no difference.” The responses were categorized into “leftward” and “rightward,” depending on whether the subject had chosen the rectangle with the darker end on the left or the right. Subjects performed a baseline session of 72 pictures without stimulation prior to the rTMS mapping session. Both sessions were videotaped for later analysis [41, 48].

### Evaluation of discomfort

After rTMS mapping, the subjects were asked to evaluate discomfort, separately for the temporal muscle area (“temporal”) and for the remainder of the head surface (“convexity”). The meter was the visual analogue scale (range 0–10): 0 signifying no pain and 10 signifying maximal pain.

### Data analysis

Data analysis comprised several steps. First we went over the subject’s video records and labeled each response as “leftward” or “rightward” (as outlined in 2.4.2). Next we related responses and stimulated cortical spots. For each spot we counted the number of effective rTMS stimulations and, among these, the number of leftward and rightward responses. Stimulation was deemed effective if a complete train of 10 rTMS pulses had been applied and if the electric field strength at the cortical level had been above 55 V/m the entire time [34]. Then scores were computed as the difference between the rightward and leftward responses divided by the number of effective stimulations (between a range of − 1.00 and 1.00). The subject’s task performance in baseline conditions was documented as the baseline score. Their performance in mapping conditions was documented for each particular spot as the particular deviation score (i.e., the spot’s computed score minus the subject’s baseline score). The deviation scores, in turn, were categorized as “leftward” or “rightward,” depending on whether the scores were negative or positive. Then we pooled the information of all the subjects per cortical spot as follows:

1. We calculated the number of subjects with leftward deviation scores and the mean of their scores, i.e., the mean of all leftward deviation scores.
2. We calculated the number of subjects with rightward deviation scores and the mean of their scores, i.e., the mean of all rightward deviation scores.

For clearer comparison, we handled all mean deviation scores in terms of their magnitude.

### Statistics

The results are listed as mean ± standard deviation plus median and range where applicable. Inter-spot comparisons were made by the Mann–Whitney U test for independent samples. For single-spot analysis (concerning “leftward” vs. “rightward” effects), we used the Wilcoxon matched-pairs signed rank test. All tests were regarded as significant at a *p* value < 0.05 (GraphPad Prism 6.0, La Jolla, CA, USA).

## Results

### Subject characteristics

The subject characteristics are listed in Table 2. We determined a mean RMT of 33.1 ± 6.4% maximal stimulator output, in terms of the left hemisphere, and of 32.9 ± 5.9%, in terms of the right hemisphere (*p* = 0.9564). Without stimulation, 9 out of 10 subjects presented with a leftward basic bias; one subject showed a rightward basic bias. Taken together, the baseline score averaged − 0.59 ± 0.51. rTMS mapping was tolerated well, and discomfort was comparable for both hemispheres. All subjects were purely right-handed and showed left-hemispheric dominance.

**Table 2:** Subject characteristics

Subject	RMT		Pain score temporal		Pain score convexity		Greyscales task baseline score
	Left hemis-phere	Right hemis-phere	Left hemis-phere	Right hemis-phere	Left hemis-phere	Right hemis-phere	
1	29	29	2	3	1	1	− 0.61
2	34	29	2	2	1	1	0.78
3	29	33	3	6	1	1	− 0.81
4	43	39	4	4	1	1	− 0.94
5	36	35	3	3	1	1	− 0.94
6	30	28	2	2	0	0	− 0.97
7	45	45	6	6	1	1	− 0.75
8	31	36	4	6	1	3	− 0.47
9	28	26	4	2	3	1	− 0.67
10	26	29	2	4	1	3	− 0.53
Mean	33.1	32.9	3.2	3.8	1.1	1.3	− 0.59
SD	6.4	5.9	1.3	1.7	0.7	0.9	0.51
Median	30.5	31	3	3.5	1	1	− 0.71
MIN	26	26	2	2	0	0	− 0.97
MAX	45	45	6	6	3	3	0.78
	*p* = 0.9564		*p* = 0.5493		*p* = 0.8375		

Subject characteristics. Resting motor threshold (RMT) as  % of stimulator output. Pain score according to the visual analogue scale (VAS), range from 0 (no pain) to 10 (maximal pain). Greyscales task baseline score determined on 72 pictures without stimulation, range from − 1.00 (leftward bias) to 1.00 (rightward bias)

### Number and size of deviations

Tables 3 and 4 provide all computed deviation scores on mapping conditions. Additional subject-related scores are available as an online resource (Additional files 1, 2). First we had a look at the number and size of leftward and rightward deviations.

**Table 3:** Deviation scores per cortical spot for the left hemisphere

Cortical spot	“Leftward” deviation scores		“Rightward” deviation scores	
	Number of subjects	Mean of these subjects’ scores	Number of subjects	Mean of these subjects’ scores
1	2	− 0.06	8	0.26
2	5	− 0.13	5	0.15
3	2	− 0.09	8	0.27
4	2	− 0.06	8	0.40
5	3	− 0.23	7	0.34
6	4	− 0.11	6	0.33
7	5	− 0.22	5	0.30
8	2	− 0.26	8	0.34
9	5	− 0.19	5	0.29
10	3	− 0.24	7	0.33
11	4	− 0.13	6	0.28
12	4	− 0.16	6	0.31
13	5	− 0.13	5	0.23
14	7	− 0.17	3	0.31
15	7	− 0.26	3	0.88
16	6	− 0.16	4	0.52
17	7	− 0.15	3	0.36
18	4	− 0.14	6	0.25
19	7	− 0.12	3	0.40
20	5	− 0.22	5	0.42
21	6	− 0.19	4	0.46
22	7	− 0.18	3	0.06
23	6	− 0.22	4	0.20
24	9	− 0.23	1	0.55
25	8	− 0.23	2	0.29
26	7	− 0.24	3	0.17
27	9	− 0.20	1	0.47
28	5	− 0.38	5	0.13
29	6	− 0.31	4	0.20
30	6	− 0.15	4	0.52
31	6	− 0.29	4	0.21
32	5	− 0.18	5	0.36
33	7	− 0.26	3	0.78
34	6	− 0.21	4	0.58
35	5	− 0.32	5	0.61
36	4	− 0.22	6	0.33
37	6	− 0.28	4	0.81
38	4	− 0.22	6	0.46
39	6	− 0.15	4	0.62
40	6	− 0.36	4	0.71
41	6	− 0.15	4	0.39
42	7	− 0.22	3	0.44
43	7	− 0.29	3	0.42
44	5	− 0.18	5	0.67
45	8	− 0.18	2	0.44
46	8	− 0.18	2	0.45
47	7	− 0.24	3	0.38
48	7	− 0.18	3	0.81
49	7	− 0.18	2	0.88
50	7	− 0.24	3	0.85
51	8	− 0.22	2	0.48
52	6	− 0.18	4	0.14
Mean	6	− 0.20	4	0.42
SD	2	− 0.07	2	0.20
MIN	2	− 0.06	1	0.06
MAX	9	− 0.38	8	0.88

Results for stimulation of the left hemisphere. Number of subjects with negative deviation scores (“leftward”) and mean of their scores. Number of subjects with positive deviation scores (“rightward”) and mean of their scores. Outline per cortical spot (no. 1–52) plus mean, standard deviation (SD), minimum (MIN), and maximum (MAX)

**Table 4:** Deviation scores per cortical spot for the right hemisphere

Cortical spot	“Leftward” deviation scores		“Rightward” deviation scores	
	Number of subjects	Mean of these subjects’ scores	Number of subjects	Mean of these subjects’ scores
1	4	− 0.21	6	0.56
2	6	− 0.29	4	0.63
3	7	− 0.22	3	0.73
4	7	− 0.24	3	0.96
5	7	− 0.18	3	0.93
6	7	− 0.17	3	0.62
7	7	− 0.24	3	0.33
8	5	− 0.40	5	0.32
9	7	− 0.26	3	1.15
10	6	− 0.28	4	0.72
11	6	− 0.38	4	0.73
12	7	− 0.27	3	0.44
13	7	− 0.34	3	1.15
14	7	− 0.26	3	0.95
15	6	− 0.23	4	0.48
16	7	− 0.33	3	0.52
17	6	− 0.34	4	0.43
18	5	− 0.40	5	0.73
19	6	− 0.23	4	0.58
20	4	− 0.30	6	0.44
21	4	− 0.23	6	0.44
22	5	− 0.26	5	0.80
23	4	− 0.18	5	0.72
24	6	− 0.21	4	0.68
25	3	− 0.19	6	0.57
26	5	− 0.22	5	0.58
27	5	− 0.25	5	0.65
28	5	− 0.21	5	0.46
29	5	− 0.27	5	0.61
30	4	− 0.23	6	0.80
31	5	− 0.23	5	0.65
32	4	− 0.28	6	0.49
33	4	− 0.26	6	0.63
34	6	− 0.20	4	1.04
35	4	− 0.35	6	0.41
36	5	− 0.20	5	0.73
37	7	− 0.24	3	0.90
38	7	− 0.18	3	0.77
39	4	− 0.21	6	0.43
40	6	− 0.23	4	0.53
41	4	− 0.36	6	0.59
42	5	− 0.21	5	0.38
43	6	− 0.15	4	1.04
44	6	− 0.24	4	0.79
45	7	− 0.33	3	0.58
46	6	− 0.19	4	0.87
47	7	− 0.26	3	1.26
48	5	− 0.38	5	0.65
49	7	− 0.22	3	0.56
50	7	− 0.26	3	0.91
51	7	− 0.27	3	0.59
52	4	− 0.16	6	0.41
Mean	6	− 0.25	4	0.67
SD	1	− 0.06	1	0.22
MIN	3	− 0.15	3	0.32
MAX	7	− 0.40	6	1.26

Results for stimulation of the right hemisphere. Number of subjects with negative deviation scores (“leftward”) and mean of their scores. Number of subjects with positive deviation scores (“rightward”) and mean of their scores. Outline per cortical spot 1–52 plus mean, standard deviation (SD), minimum (MIN), and maximum (MAX)

#### Leftward deviations

**Fig. 3:**
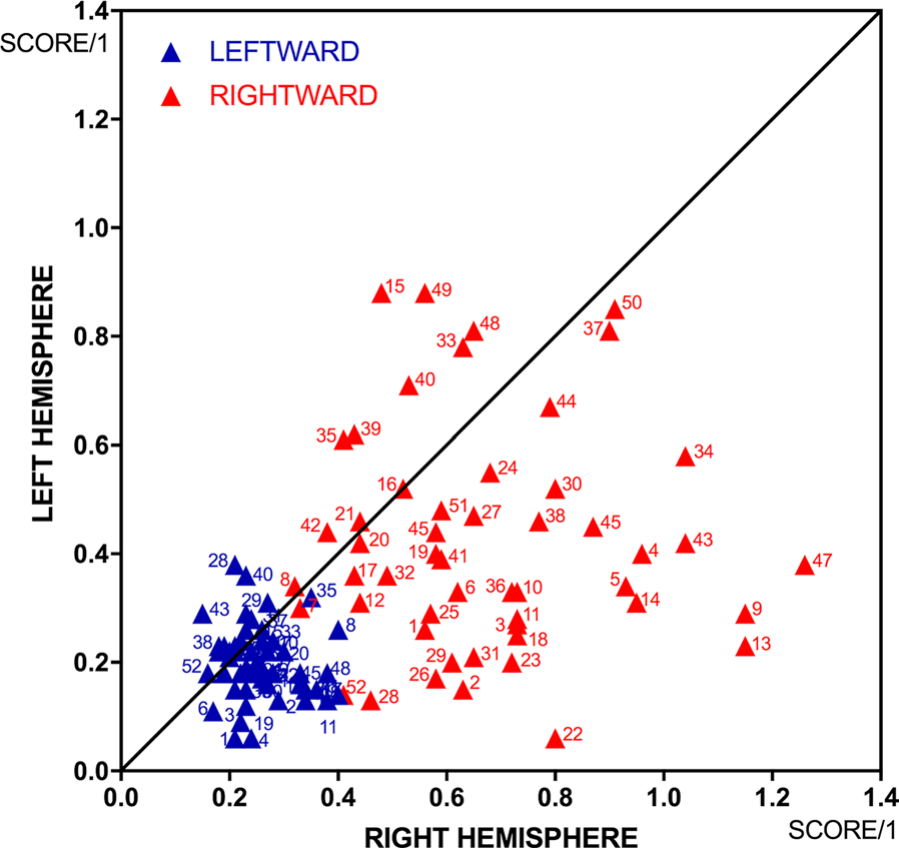
Inter-hemispheric comparison of deviations. Deviation sizes in comparison. Plotted are mean deviation scores per cortical spot (no. 1–52), as always, for the left hemisphere (y-coordinate; Table 3) and the right hemisphere (x-coordinate; Table 4). Leftward deviations in blue, rightward deviations in red

Regarding the frequency, leftward deviations occurred significantly more often than rightward deviations within both the left (*p* = 0.0077) and the right hemisphere (*p* < 0.0001). Analyzing the results of both hemispheres together, we found that inter-hemispherically, their number was comparable (*p* = 0.6397). Regarding the effect size, rTMS of the right hemisphere elicited significantly stronger leftward deviations than rTMS of the left hemisphere (*p* < 0.0001; Fig. 3). Altogether, i.e. for both hemispheres, the mean leftward deviation scores ranged from 0.06 to 0.40 in magnitude.

#### Rightward deviations

Consequently, rightward deviations were more rarely observed than leftward deviations. Their number was comparable for the two hemispheres (*p* = 0.6352). However, rightward deviations were strikingly greater in magnitude, namely, compared to leftward deviations (*p* < 0.0001) and according to inter-hemispheric comparison of the right rather than the left hemisphere (*p* < 0.0001; Fig. 3). The mean rightward deviation scores ranged from 0.06 to 1.26 in magnitude.

### Cortical distribution of deviations

**Fig. 4:**
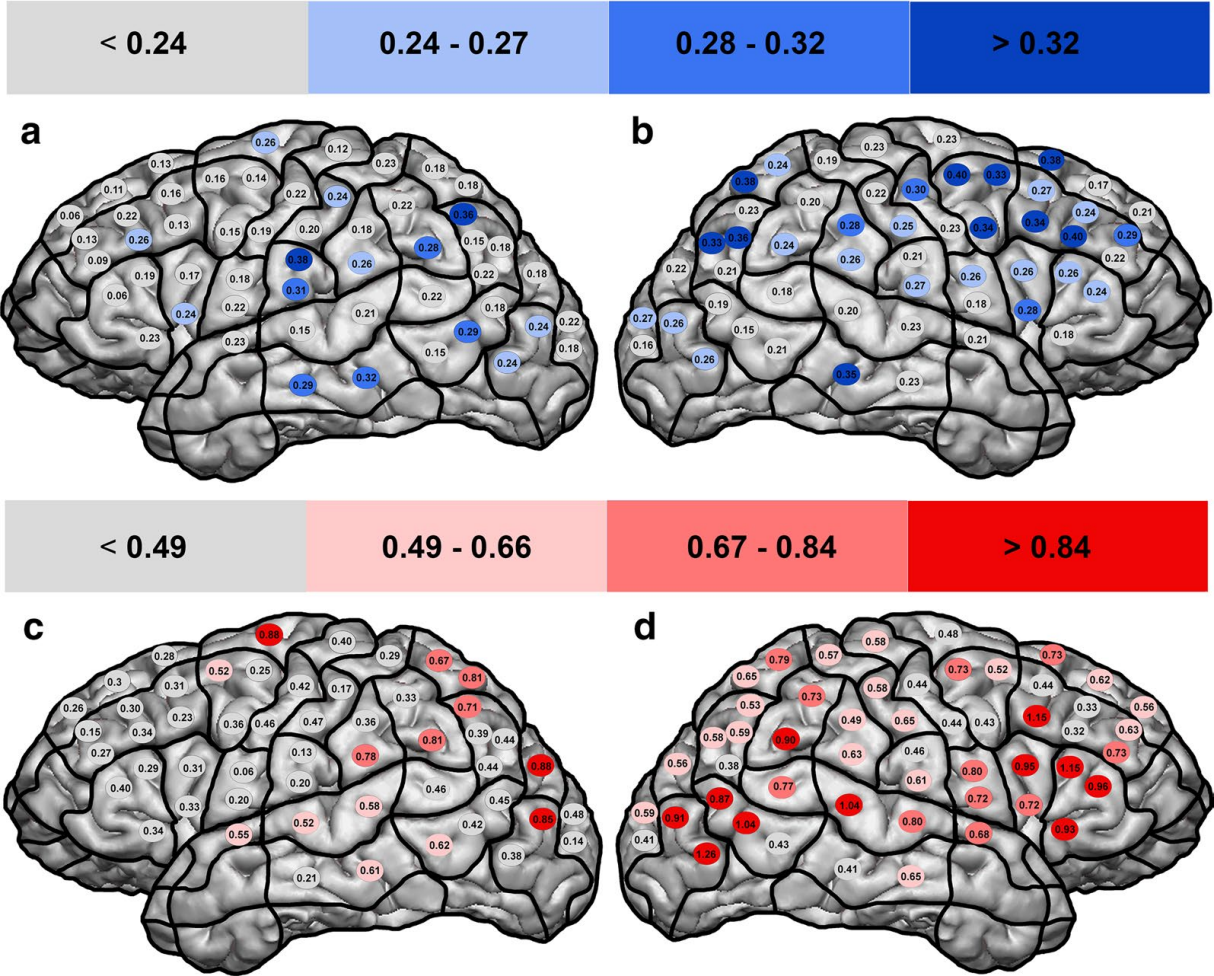
Cortical distribution of deviations. Cortical distributions within the left hemisphere (**a**, **c**) and the right hemisphere (**b**, **d**). Presented are the mean deviation scores (Tables 3, 4). Leftward deviations in blue, rightward deviations in red

In what follows we outline the cortical distribution of deviations. Figure 4 depicts the leftward deviations in blue color and the rightward deviations in red.

#### Leftward deviations

Regarding the left hemisphere, we observed strong leftward effects within parietal areas (vPoG, anG; spots no. 28, 40; Fig. 4a). Regarding the right hemisphere, the parietal areas (SPL, anG; spots no. 41, 45, 48) were as prominent as the middle middle temporal gyrus (mMTG; spot no. 35), and as a wide frontal area (mSFG, mMFG, pMFG; spots no. 8, 11, 13, 16–18, Fig. 4b).

#### Rightward deviations

Rightward deviations within the left hemisphere were distributed to the posterior superior frontal gyrus (pSFG; spot no. 15) and to occipital areas (dLOG, vLOG; spots no. 49–50; Fig. 4c). The right hemisphere showed a number of striking spots, including the posterior supramarginal gyrus (pSMG; spot no. 37), the ventral lateral occipital gyrus (vLOG; spots no. 47, 50), temporal areas (mSTG, pMTG; spots no. 34, 43, 46), and frontal areas (mMFG, trIFG, opIFG; spots no. 4, 5, 9, 13–14; Fig. 4d).

### Raw data

We provide our subjects’ raw data as an online resource (Additional files 3, 4). Cortical spots of the left hemisphere were stimulated 9.8 ± 0.2 times on average, and cortical spots of the right hemisphere were stimulated 9.7 ± 0.3 times. The number of effective stimulations per spot ranged from 5 to 15 for the left hemisphere and from 4 to 12 for the right hemisphere.

## Discussion

### General aims and limitations

We have already reported on the usability of navigated rTMS to mimic visual neglect and map corresponding cortical areas in another study [36]. While searching for preferably sensitive visuospatial tasks, we also came across literature on the greyscales task and, thus, designed the pilot study presented in this manuscript. We mainly focused on general feasibility and the broad-ranging examination of both hemispheres, which involved accepting a number of limitations. To assess new setups and to understand the anatomical correlates of pathologies, it is crucial to examine healthy subjects. Our volunteers formed a small and homogenous healthy cohort, which may be seen as a benefit [49]. At the same time, it may be seen as a restriction, and the generalizability of our findings certainly must be further assessed in relation to a higher number of subjects of all ages. Moreover, we should be aware of limitations due to our rTMS protocol. We tested a wide range of brain areas using a fixed mapping template. We stimulated at a frequency of 5 Hz and with strict anterior–posterior field orientation. Several protocol changes, for example, varying coil angulations, could have modified our results [50]. However, we proceeded comparably to all current mapping standards that have been used before [36, 37, 45]. Some cortical spots showed a quite small number of effective stimulations. However, the mean number of stimulations per spot was over 9.1 for all subjects with a consistently small variance. As a last point, we can neither offer any test–retest evaluation in the form of a second examination, nor any sham-stimulation controls to exclude factors such as concentration deficits or unintended remote rTMS effects. This should be the next step following this feasibility study. With all this in mind, our findings should clearly be carefully considered. Nevertheless, as a first step in an evaluation, we may rate them as useful and encouraging for a further pursuit of this approach.

### The greyscales task

Neglect patients are known to develop various mechanisms to compensate for existent pathologies. Hence, a true diagnosis requires precise and challenging task selections [26]. The greyscales task serves as a sensitive tool to measure perceptual biases in healthy subjects and in patients and has even been used to uncover deficits in patients without apparent visual neglect in conservative testing [27, 30]. In this study we chose a computerized and tachistoscopic application and conclusively can approve this setting. It proved to be applicable and highly sensitive. Tachistoscopic task display prevents effects such as fixation or eye scanning. As originally conducted, our subjects had to respond verbally. We had to take into account the fact that left-hemisphere-activation by speaking might affect the inter-hemispheric processes of visuospatial attention. On the other hand manual demands have also been reported as affecting results—for example, depending on the hand being used to perform [18]. A key advantage of the greyscales task is that there are no errors to make or be detected, but each response contributes to the overall result, representing the subject’s fully individual tendency with regard to visuospatial attention processing. By determining a basic bias prior to the rTMS examination and considering all subsequent results in relation to this value, there is no usability limitation accompanying the already existent deficits. Here we examined a collective of healthy men, but our setting may be applied to patients as well. Moreover, the adaptability does not depend on the presence or form of pseudoneglect. Our baseline findings are consistent with reports on the prevalence of pseudoneglect among young adults: 9 out of our 10 subjects naturally tended to the left rather than the right [18, 20, 22, 27]. With advancing age, pseudoneglect is known to shift rightwards [51, 52]. This fact should be kept in mind for future analysis of patient data, but as stated above, it does not restrict the applicability. Besides, we should mention that Friedrich et al. analyzed the age factor of pseudoneglect by means of the greyscales task and found that healthy elderly people presented with an even stronger leftward bias than their younger participants [53].

### rTMS mapping

Across the literature, visuospatial attention is described as highly individually distributed, balanced, and suggestible [1, 54–56]. However, we assume that a scaffold of cortical spots exists connected anatomically, that they are thus available by order of visuospatial function, and that they are at least available to be recruited if necessary. As already addressed in 4.1, our rTMS results certainly should not be considered absolute. There were cortical spots with outstanding deviation scores averaged over less than half of our subjects; the other subjects were either not suggestible (but by chance showed small deviation scores in the opposite direction) or alternatively were suggestible but, as a matter of fact, in the opposite direction (Tables 3, 4; Additional files 1, 2, 3, 4). One more factor we should mention is the experiment’s fairly long time span. A natural leftward bias on baseline performance is known to decline in the course of visuospatial task demands. Due to diminished alertness and neural fatigue, biases shift rightward naturally over time [57–59]. We examined the two hemispheres in the order left–right-left–right, i.e., in two turns. To prevent a time-on-task effect, we took breaks after every examination of one hemisphere, and we periodically animated our subjects to maintain concentration for the time span in between. An increasing rightward shift over time should have resulted in a higher total number of rightward deviations for the right hemisphere compared to the left hemisphere. Fortunately, we could not find any pattern of time-effects. The number of rightward deviations was comparable for both hemispheres (see “Rightward deviations” section).

To get a better measure of our findings, we performed a principal analysis of deviation numbers and sizes, as outlined in 3.2. Leftward deviations were recorded significantly more often and were significantly smaller in magnitude than rightward deviations. The higher frequency may be based on pre-existent pseudoneglect and might solely reflect right hemisphere activity during visuospatial task demands, especially as the score values tended to be small. On the other hand, an already leftward baseline score limited the attainable magnitude of negative deviation scores per se. In contrast, rightward deviations were found to be strikingly great in magnitude and significantly stronger than leftward deviations (Fig. 3; Tables 3, 4). Once more referring to the baseline performance, we could categorize these rightward deviations as a reduction or cancellation of the natural leftward bias, i.e., of pseudoneglect. This pseudoneglect “ceiling effect” has been described before [14, 17]. Furthermore these rightward deviations parallel the classic symptom of left visual neglect. In clinical routine, visual neglect is described as being both the most common and most pronounced phenomenon after right hemispheric damage [7, 8, 27, 60]. Accordingly, we found the right hemisphere to be significantly more suggestible by rTMS than the left hemisphere (Figs. 3, 4). This is also in line with our initial assumption that rTMS of the right hemisphere ought to strikingly misbalance the base state of processing in which the right hemisphere takes the dominant part. To summarize, we may reaffirm that rTMS affords a useful opportunity to map visuospatial attention function at the cortical level, most convincingly for the right hemisphere and—when examining healthy men with pseudoneglect—for attention processing to the right.

### Cortical distributions with reference to the current literature

The unquestionably best-known form of visual neglect is the combination of right hemispheric parietal damage followed by contralesional left deficits. Notwithstanding, there are more and more reports of other lesion locations and clinical manifestations, up to reports on the concurrent occurrence of ipsi- and contralesional deficits [47, 61–64]. As a side note, the greyscales study by Mattingley et al. [27] also included two right-parietal patients with an extreme leftward bias and thus ipsilesional neglect. As introduced above, studies on pseudoneglect in healthy adults have additionally helped to explain processing mechanisms [14–18, 20, 22–25]. However, a comparative discussion of results proves difficult because of the heterogeneity of approaches. Studies use different tasks to measure visuospatial deficits, focus on different locations, and interpret their results from different angles. One fact upon which they all agree, which has persisted over the course of decades, is that the right hemisphere at least plays a somewhat special role, whether dominant or controlling [65, 66]. This idea also provides the basis for explaining the high prevalence of pseudoneglect in healthy adults [15, 16, 21–24, 51]. Regarding cortical distributions, there is the widely accepted idea of subcortical fiber tracts connecting frontal areas with parietal areas and the temporoparietal junction (TPJ) [2, 3, 24, 54, 55, 65, 67]. Corbetta and Shulman assume two networks: a dorsal network including superior parietal and frontal areas represented on both hemispheres, and a ventral network including the TPJ and inferior frontal areas represented dominantly on the right hemisphere and supervising the dorsal network [3]. To class our findings with these models, we have to differentiate between the left and the right hemisphere. Within the left hemisphere occasional spots of frontal, parietal, and lateral occipital areas presented with strong deviation effects (Fig. 4a, c), though we cannot distribute them distinctly to the stated networks and must suggest forming careful conclusions from these findings. Yet, rTMS-lesioning of the right hemisphere detected cortical spots that accorded well with the introduced models. Interestingly, we found leftward deviations (corresponding to ipsilesional neglect) to mainly be distributed to posterior parietal and superior frontal areas, according to the proposed dorsal network (Fig. 4b; Table 4). The observation of leftward instead of rightward deviations does not go in line with the basic responsibilities Corbetta and Shulmann intended for their networks [3]. However, supposing equal neuronal structures and thus rTMS-effects for dorsal or ventral brain regions, we should contemplate subtler task allocations within the dorsal network. There are several publications on the occurrence of ipsilesional neglect after right-hemispheric damage [62, 63, 68, 69]. Chokron et al. [70] even reported right visual neglect in patients with left hemianopia plus neglect. Especially the role of frontal and subcortical areas is discussed, albeit, so far, there is no generally accepted explanation that could be integrated into the model of Corbetta and Shulmann [61, 64]. On the contrary, rightward deviations (corr. to contralesional neglect) could be triggered best at inferior frontal spots and at a pool of spots within the area of the TPJ (Fig. 4d; Table 4). In turn, these observations comply with both localization and function of a ventral network.

At this point we also want to mention our group’s first work on neglect, which was a combination of rTMS and a classical landmark task [36]. We successfully showed the feasibility of mapping visuospatial attention, yet the landmark task solely provided information in the form of right-or-wrong answers, and the resulting error rates among our healthy volunteers tended to be rather small. The study presented here can be seen as a second approach to gather more and better comparable data using the greyscales task. As already outlined, the greyscales task takes into account any recorded answer and allows interpretations independently from any existent deficits. Since the two tasks use quite different ways of measuring visuospatial attention and respectively different forms of analysis, and since both approaches conformed to pilot studies’ inclusive limitations, we decided not to compare single results. However, we may summarize that the findings of both go well together, embedded in the generally acknowledged model of visuospatial processing. Regarding the right hemisphere, we found consistent distributions in the area of the TPJ and for spots of the middle frontal gyrus. For clinical purposes the greyscales task design stands out by being quite easily applicable and bearable while achieving sensitive results. To reach similar sensitivity for the landmark task, we would have had to increase its difficulty, for example, by shortening the line differences between the left and right segments. Yet, all our healthy subjects reported the landmark task as being particularly demanding, which is why we seriously doubt its feasibility at a higher difficulty level, let alone in elderly patients.

### Future prospects

Obviously, the acting and interacting of networks responsible for visuospatial attention has not yet been understood to the fullest extent. Research increasingly concentrates on the subcortical level [71–73]. However, several options are conceivable to integrate cortical mapping using rTMS. For example, a combination with fMRI enables the detection of unintended remote stimulation effects and potentially accountable white matter connections [74]. Furthermore, seminal approaches are made by diffusion tensor imaging fiber tracking. The combination of diffusion tensor imaging fiber tracking and rTMS language mapping recently obtained highly promising results for the imaging of subcortical language pathways and may be assessed similarly for the rTMS-mapped visuospatial attention function [75–78]. Basic research naturally aims to yield a clinical advantage. It could be shown that neurosurgeons profit by presurgical maps by preventing functional deficits while allowing maximal resection [9, 79]. In patients with certain tumor locations, we should consider adding maps of visuospatial attention function to the individual preoperative assessment. On the other hand, dealing with already existent deficits, neurologists currently develop new treatment regimes. In light of visual neglect being the result of damage accompanied by a misbalancing of large-scale brain networks, recovery correlates with rebalancing [1, 11, 80]. Once more, the presented combination of the greyscales task and rTMS may be advantageous in terms of generating individual and accurate cortical maps for therapeutic interventions.

## Conclusion

Referring to our initial hypotheses, we can conclude that the greyscales task on tachistoscopic test conditions, in combination with rTMS, is appropriate, sensitive, and accurate in mapping visuospatial attention function on a cortical level.

## Additional files

**Additional file 1.** Subject-related deviation scores per cortical spot for the left hemisphere. Results for stimulation of the left hemisphere. Deviation scores of subject 1–10. Number of subjects with negative deviation scores (“leftward”) and mean of their scores. Number of subjects with positive deviation scores (“rightward”) and mean of their scores. Outline per cortical spot (no. 1–52) plus mean, standard deviation (SD), minimum (MIN), and maximum (MAX).

**Additional file 2.** Subject-related deviation scores per cortical spot for the right hemisphere Results for stimulation of the right hemisphere. Deviation scores of subject 1–10. Number of subjects with negative deviation scores (“leftward”) and mean of their scores. Number of subjects with positive deviation scores (“rightward”) and mean of their scores. Outline per cortical spot (no. 1–52) plus mean, standard deviation (SD), minimum (MIN), and maximum (MAX).

**Additional file 3.** Raw data per cortical spot for the left hemisphere. Results for stimulation of the left hemisphere. Raw data of each subject. Number of effective stimulations, “leftward” answers and “rightward” answers. Outline per cortical spot (no. 1–52) plus mean, standard deviation (SD), minimum (MIN), and maximum (MAX).

**Additional file 4.** Raw data per cortical spot for the right hemisphere. Results for stimulation of the right hemisphere. Raw data of each subject. Number of effective stimulations, “leftward” answers and “rightward” answers. Outline per cortical spot (no. 1–52) plus mean, standard deviation (SD), minimum (MIN), and maximum (MAX).

## Abbreviations

fMRI: functional magnetic resonance imaging
MRI: magnetic resonance imaging
RMT: resting motor threshold
rTMS: repetitive navigated transcranial magnetic stimulation
TMS: transcranial magnetic stimulation
TPJ: temporoparietal junction
