# Supplementary material for: Mapping visuospatial attention: the greyscales task in combination with repetitive navigated transcranial magnetic stimulation
Source: BMC Neurosci. 2018 Jul 11;19:40. doi: 10.1186/s12868-018-0440-1 (PMC6042394; doi:10.1186/s12868-018-0440-1)
Supplement: Supplementary file 2 — Additional file 2. Subject-related deviation scores per cortical spot for the right hemisphere Results for stimulation of the right hemisphere. Deviation scores of subject 1–10. Number of subjects with negative deviation scores (“leftward”) and mean of their scores. Number of subjects with positive deviation scores (“rightward”) and mean of their scores. Outline per cortical spot (no. 1–52) plus mean, standard deviation (SD), minimum (MIN), and maximum (MAX). [file 12868_2018_440_MOESM2_ESM.docx]

**Manuscript Number:**

**Article Title: Mapping visuospatial attention – the greyscales task in combination with repetitive navigated transcranial magnetic stimulation**

**Journal Name:**

**Authors: Katrin Giglhuber; Stefanie Maurer; Claus Zimmer, MD; Bernhard Meyer, MD; Sandro M. Krieg, MD, MBA**

**Correspondence: Sandro.Krieg@tum.de**

**Additional file 2:** Subject-related deviation scores per cortical spot for the right hemisphere

| cortical spot | deviation scores | | | | | | | | | | | | | |
| --- | --- | --- | --- | --- | --- | --- | --- | --- | --- | --- | --- | --- | --- | --- |
|  | subject | | | | | | | | | | “leftward” | | “rightward” | |
|  | 1 | 2 | 3 | 4 | 5 | 6 | 7 | 8 | 9 | 10 | number | mean | number | mean |
| 1 | -0.19 | 0.22 | 1.41 | -0.06 | -0.06 | 0.37 | 0.95 | -0.53 | 0.27 | 0.13 | 4 | -0.21 | 6 | 0.56 |
| 2 | -0.39 | -0.58 | 1.58 | -0.06 | -0.06 | 0.17 | 0.55 | -0.53 | -0.13 | 0.19 | 6 | -0.29 | 4 | 0.63 |
| 3 | -0.19 | -0.38 | 1.58 | -0.06 | -0.06 | -0.03 | 0.55 | -0.53 | 0.07 | -0.27 | 7 | -0.22 | 3 | 0.73 |
| 4 | -0.39 | 0.02 | 1.31 | -0.06 | -0.06 | -0.03 | 1.55 | -0.33 | -0.33 | -0.47 | 7 | -0.24 | 3 | 0.96 |
| 5 | -0.17 | 0.22 | 1.56 | -0.06 | -0.06 | -0.03 | 1.00 | -0.53 | -0.13 | -0.27 | 7 | -0.18 | 3 | 0.93 |
| 6 | -0.19 | -0.18 | 1.14 | -0.06 | -0.06 | 0.17 | 0.55 | -0.53 | -0.13 | -0.07 | 7 | -0.17 | 3 | 0.62 |
| 7 | -0.19 | -0.67 | 0.06 | -0.06 | -0.06 | 0.17 | 0.75 | -0.13 | -0.33 | -0.27 | 7 | -0.24 | 3 | 0.33 |
| 8 | -0.19 | -0.78 | 0.47 | 0.14 | -0.06 | 0.37 | 0.55 | -0.53 | 0.07 | -0.47 | 5 | -0.40 | 5 | 0.32 |
| 9 | -0.39 | -0.18 | 1.36 | -0.06 | -0.06 | 0.57 | 1.53 | -0.53 | -0.33 | -0.27 | 7 | -0.26 | 3 | 1.15 |
| 10 | -0.19 | -0.58 | 0.47 | -0.06 | -0.06 | 1.37 | 0.75 | -0.33 | 0.27 | -0.47 | 6 | -0.28 | 4 | 0.72 |
| 11 | -0.19 | -0.98 | 0.92 | -0.06 | -0.06 | 0.77 | 1.15 | -0.53 | 0.07 | -0.47 | 6 | -0.38 | 4 | 0.73 |
| 12 | -0.19 | -0.67 | 0.21 | -0.06 | -0.06 | 0.37 | 0.75 | -0.53 | -0.13 | -0.27 | 7 | -0.27 | 3 | 0.44 |
| 13 | -0.39 | -0.58 | 1.14 | -0.06 | -0.06 | 1.37 | 0.95 | -0.53 | -0.33 | -0.47 | 7 | -0.34 | 3 | 1.15 |
| 14 | -0.39 | -0.18 | 0.92 | -0.06 | -0.06 | 0.97 | 0.95 | -0.53 | -0.13 | -0.47 | 7 | -0.26 | 3 | 0.95 |
| 15 | -0.39 | -0.06 | 0.66 | -0.06 | -0.06 | 0.64 | 0.55 | -0.33 | 0.07 | -0.47 | 6 | -0.23 | 4 | 0.48 |
| 16 | -0.39 | -0.44 | 0.25 | -0.06 | -0.06 | 0.37 | 0.95 | -0.53 | -0.33 | -0.47 | 7 | -0.33 | 3 | 0.52 |
| 17 | -0.39 | -0.78 | -0.01 | -0.06 | 0.34 | 0.97 | 0.35 | -0.53 | 0.07 | -0.27 | 6 | -0.34 | 4 | 0.43 |
| 18 | -0.19 | -0.78 | 1.31 | -0.06 | 0.14 | 0.77 | 1.35 | -0.53 | 0.07 | -0.47 | 5 | -0.40 | 5 | 0.73 |
| 19 | 0.21 | -0.35 | 0.81 | -0.06 | -0.06 | 0.57 | 0.75 | -0.53 | -0.13 | -0.27 | 6 | -0.23 | 4 | 0.58 |
| 20 | 0.21 | 0.22 | 0.41 | -0.06 | 0.17 | 1.08 | 0.55 | -0.53 | -0.13 | -0.47 | 4 | -0.30 | 6 | 0.44 |
| 21 | 0.01 | 0.22 | 0.61 | -0.06 | -0.06 | 0.97 | 0.55 | -0.53 | 0.27 | -0.27 | 4 | -0.23 | 6 | 0.44 |
| 22 | -0.19 | 0.22 | 1.01 | -0.06 | -0.06 | 0.97 | 1.55 | -0.53 | 0.27 | -0.47 | 5 | -0.26 | 5 | 0.80 |
| 23 | 0.61 | 0.00 | 0.61 | -0.06 | -0.06 | 1.17 | 0.75 | -0.33 | 0.47 | -0.27 | 4 | -0.18 | 5 | 0.72 |
| 24 | 0.21 | -0.22 | 1.01 | -0.06 | -0.06 | 0.97 | 0.55 | -0.33 | -0.13 | -0.47 | 6 | -0.21 | 4 | 0.68 |
| 25 | 0.81 | 0.00 | 0.95 | -0.06 | -0.06 | 0.97 | 0.55 | 0.07 | 0.07 | -0.47 | 3 | -0.19 | 6 | 0.57 |
| 26 | -0.19 | 0.22 | 1.08 | -0.06 | -0.06 | 0.97 | 0.55 | -0.53 | 0.07 | -0.27 | 5 | -0.22 | 5 | 0.58 |
| 27 | 0.01 | 0.02 | 0.92 | -0.06 | -0.06 | 0.97 | 1.35 | -0.53 | -0.13 | -0.47 | 5 | -0.25 | 5 | 0.65 |
| 28 | 0.50 | 0.22 | 0.47 | -0.06 | -0.06 | 0.97 | 0.15 | -0.53 | -0.13 | -0.27 | 5 | -0.21 | 5 | 0.46 |
| 29 | 0.61 | -0.22 | 0.47 | -0.06 | -0.06 | 0.97 | 0.75 | -0.53 | 0.27 | -0.47 | 5 | -0.27 | 5 | 0.61 |
| 30 | 0.21 | 0.22 | 1.58 | -0.06 | -0.06 | 0.97 | 1.75 | -0.53 | 0.07 | -0.27 | 4 | -0.23 | 6 | 0.80 |
| 31 | -0.19 | 0.02 | 1.14 | 0.17 | -0.06 | 0.97 | 0.95 | -0.53 | -0.11 | -0.27 | 5 | -0.23 | 5 | 0.65 |
| 32 | 0.21 | 0.02 | 0.92 | -0.06 | -0.06 | 0.97 | 0.35 | -0.53 | 0.47 | -0.47 | 4 | -0.28 | 6 | 0.49 |
| 33 | -0.19 | 0.22 | 0.81 | 0.17 | -0.06 | 0.97 | 1.35 | -0.33 | 0.27 | -0.47 | 4 | -0.26 | 6 | 0.63 |
| 34 | -0.19 | 0.22 | 1.21 | -0.06 | -0.06 | 1.17 | 1.55 | -0.53 | -0.11 | -0.27 | 6 | -0.20 | 4 | 1.04 |
| 35 | 0.01 | 0.22 | 0.92 | 0.14 | -0.06 | 0.97 | 0.19 | -0.53 | -0.33 | -0.47 | 4 | -0.35 | 6 | 0.41 |
| 36 | -0.39 | -0.22 | 1.61 | -0.06 | -0.06 | 0.97 | 0.95 | 0.07 | 0.07 | -0.27 | 5 | -0.20 | 5 | 0.73 |
| 37 | -0.39 | 0.22 | 0.92 | -0.06 | -0.06 | -0.03 | 1.55 | -0.53 | -0.33 | -0.27 | 7 | -0.24 | 3 | 0.90 |
| 38 | -0.19 | 0.22 | 1.14 | -0.06 | -0.06 | -0.03 | 0.95 | -0.13 | -0.33 | -0.47 | 7 | -0.18 | 3 | 0.77 |
| 39 | -0.19 | 0.02 | 0.41 | -0.06 | 0.14 | 0.97 | 0.95 | 0.07 | -0.33 | -0.27 | 4 | -0.21 | 6 | 0.43 |
| 40 | -0.39 | 0.02 | 1.41 | -0.06 | 0.14 | -0.03 | 0.55 | -0.53 | -0.13 | -0.27 | 6 | -0.23 | 4 | 0.53 |
| 41 | -0.39 | 0.22 | 1.21 | -0.06 | 0.34 | 0.17 | 1.15 | -0.53 | 0.47 | -0.47 | 4 | -0.36 | 6 | 0.59 |
| 42 | -0.19 | -0.22 | 0.56 | -0.06 | 0.34 | 0.17 | 0.55 | -0.53 | 0.27 | -0.07 | 5 | -0.21 | 5 | 0.38 |
| 43 | -0.19 | -0.18 | 0.92 | -0.06 | -0.06 | 1.97 | 1.15 | 0.14 | -0.33 | -0.07 | 6 | -0.15 | 4 | 1.04 |
| 44 | -0.39 | 0.22 | 0.61 | -0.06 | -0.06 | 0.97 | 1.35 | -0.53 | -0.13 | -0.27 | 6 | -0.24 | 4 | 0.79 |
| 45 | -0.39 | -0.89 | 0.47 | 0.49 | -0.06 | 0.77 | -0.05 | -0.53 | -0.33 | -0.07 | 7 | -0.33 | 3 | 0.58 |
| 46 | -0.39 | -0.18 | 0.47 | -0.06 | -0.06 | 1.77 | 0.95 | -0.13 | -0.33 | 0.28 | 6 | -0.19 | 4 | 0.87 |
| 47 | -0.39 | -0.18 | 1.36 | -0.06 | -0.06 | 1.77 | 0.64 | -0.33 | -0.33 | -0.47 | 7 | -0.26 | 3 | 1.26 |
| 48 | 0.21 | -0.98 | 1.06 | -0.06 | -0.06 | 0.57 | 1.15 | -0.53 | 0.27 | -0.27 | 5 | -0.38 | 5 | 0.65 |
| 49 | -0.39 | -0.38 | 0.61 | -0.06 | -0.06 | -0.03 | 0.95 | -0.33 | -0.33 | 0.13 | 7 | -0.22 | 3 | 0.56 |
| 50 | -0.39 | -0.38 | 1.01 | -0.06 | -0.06 | 0.97 | 0.75 | -0.33 | -0.33 | -0.27 | 7 | -0.26 | 3 | 0.91 |
| 51 | -0.39 | 0.02 | 1.01 | -0.06 | -0.06 | -0.03 | 0.75 | -0.53 | -0.33 | -0.47 | 7 | -0.27 | 3 | 0.59 |
| 52 | -0.19 | 0.22 | 1.41 | -0.06 | -0.06 | 0.17 | 0.35 | -0.33 | 0.27 | 0.03 | 4 | -0.16 | 6 | 0.41 |
| Mean | -0.14 | -0.16 | 0.91 | -0.03 | -0.02 | 0.71 | 0.86 | -0.42 | -0.05 | -0.30 | 6 | -0.25 | 4 | 0.67 |
| SD | 0.30 | 0.37 | 0.42 | 0.09 | 0.11 | 0.51 | 0.41 | 0.19 | 0.25 | 0.19 | 1 | -0.06 | 1 | 0.22 |
| MIN | -0.39 | -0.98 | -0.01 | -0.06 | -0.06 | -0.03 | -0.05 | -0.53 | -0.33 | -0.47 | 3 | -0.15 | 3 | 0.32 |
| MAX | 0.81 | 0.22 | 1.61 | 0.49 | 0.34 | 1.97 | 1.75 | 0.14 | 0.47 | 0.28 | 7 | -0.40 | 6 | 1.26 |
