# Supplementary material for: Mapping visuospatial attention: the greyscales task in combination with repetitive navigated transcranial magnetic stimulation
Source: BMC Neurosci. 2018 Jul 11;19:40. doi: 10.1186/s12868-018-0440-1 (PMC6042394; doi:10.1186/s12868-018-0440-1)
Supplement: Supplementary file 4 — Additional file 4. Raw data per cortical spot for the right hemisphere. Results for stimulation of the right hemisphere. Raw data of each subject. Number of effective stimulations, “leftward” answers and “rightward” answers. Outline per cortical spot (no. 1–52) plus mean, standard deviation (SD), minimum (MIN), and maximum (MAX). [file 12868_2018_440_MOESM4_ESM.docx]

**Manuscript Number:**

**Article Title: Mapping visuospatial attention – the greyscales task in combination with repetitive navigated transcranial magnetic stimulation**

**Journal Name:**

**Authors: Katrin Giglhuber; Stefanie Maurer; Claus Zimmer, MD; Bernhard Meyer, MD; Sandro M. Krieg, MD, MBA**

**Correspondence: Sandro.Krieg@tum.de**

**Additional file 4:** Raw data per cortical spot for the right hemisphere

| subject 1 | | | |
| --- | --- | --- | --- |
| cortical spot | number of  effective stimulations | number of  “leftward” answers | number of  “rightward” answers |
| 1 | 10 | 9 | 1 |
| 2 | 10 | 10 | 0 |
| 3 | 10 | 9 | 1 |
| 4 | 10 | 10 | 0 |
| 5 | 9 | 8 | 1 |
| 6 | 10 | 9 | 1 |
| 7 | 10 | 9 | 1 |
| 8 | 10 | 9 | 1 |
| 9 | 10 | 10 | 0 |
| 10 | 10 | 9 | 1 |
| 11 | 10 | 9 | 1 |
| 12 | 10 | 9 | 1 |
| 13 | 10 | 10 | 0 |
| 14 | 10 | 10 | 0 |
| 15 | 10 | 10 | 0 |
| 16 | 10 | 10 | 0 |
| 17 | 10 | 10 | 0 |
| 18 | 10 | 9 | 1 |
| 19 | 10 | 7 | 3 |
| 20 | 10 | 7 | 3 |
| 21 | 10 | 8 | 2 |
| 22 | 10 | 9 | 1 |
| 23 | 10 | 5 | 5 |
| 24 | 10 | 7 | 3 |
| 25 | 10 | 4 | 6 |
| 26 | 10 | 9 | 1 |
| 27 | 10 | 8 | 2 |
| 28 | 9 | 5 | 4 |
| 29 | 10 | 5 | 5 |
| 30 | 10 | 7 | 3 |
| 31 | 10 | 9 | 1 |
| 32 | 10 | 7 | 3 |
| 33 | 10 | 9 | 1 |
| 34 | 10 | 9 | 1 |
| 35 | 10 | 8 | 2 |
| 36 | 10 | 10 | 0 |
| 37 | 10 | 10 | 0 |
| 38 | 10 | 9 | 1 |
| 39 | 10 | 9 | 1 |
| 40 | 10 | 10 | 0 |
| 41 | 10 | 10 | 0 |
| 42 | 10 | 9 | 1 |
| 43 | 10 | 9 | 1 |
| 44 | 10 | 10 | 0 |
| 45 | 10 | 10 | 0 |
| 46 | 10 | 10 | 0 |
| 47 | 10 | 10 | 0 |
| 48 | 10 | 7 | 3 |
| 49 | 9 | 9 | 0 |
| 50 | 10 | 10 | 0 |
| 51 | 10 | 10 | 0 |
| 52 | 10 | 9 | 1 |
| Mean | 9.9 | 8.7 | 1.2 |
| SD | 0.2 | 1.5 | 1.5 |
| MIN | 9 | 4 | 0 |
| MAX | 10 | 10 | 6 |

| subject 2 | | | |
| --- | --- | --- | --- |
| cortical spot | number of  effective stimulations | number of  “leftward” answers | number of  “rightward” answers |
| 1 | 11 | 0 | 11 |
| 2 | 10 | 4 | 6 |
| 3 | 10 | 3 | 7 |
| 4 | 10 | 1 | 9 |
| 5 | 5 | 0 | 5 |
| 6 | 10 | 2 | 8 |
| 7 | 9 | 4 | 5 |
| 8 | 10 | 5 | 5 |
| 9 | 10 | 2 | 8 |
| 10 | 10 | 4 | 6 |
| 11 | 10 | 6 | 4 |
| 12 | 9 | 4 | 5 |
| 13 | 10 | 4 | 6 |
| 14 | 10 | 2 | 8 |
| 15 | 7 | 1 | 6 |
| 16 | 9 | 3 | 6 |
| 17 | 8 | 4 | 4 |
| 18 | 10 | 5 | 5 |
| 19 | 7 | 2 | 5 |
| 20 | 10 | 0 | 10 |
| 21 | 9 | 0 | 9 |
| 22 | 10 | 0 | 10 |
| 23 | 9 | 1 | 8 |
| 24 | 9 | 2 | 7 |
| 25 | 9 | 1 | 8 |
| 26 | 10 | 0 | 10 |
| 27 | 10 | 1 | 9 |
| 28 | 10 | 0 | 10 |
| 29 | 9 | 2 | 7 |
| 30 | 10 | 0 | 10 |
| 31 | 10 | 1 | 9 |
| 32 | 10 | 1 | 9 |
| 33 | 10 | 0 | 10 |
| 34 | 10 | 0 | 10 |
| 35 | 10 | 0 | 10 |
| 36 | 9 | 2 | 7 |
| 37 | 10 | 0 | 10 |
| 38 | 10 | 0 | 10 |
| 39 | 10 | 1 | 9 |
| 40 | 10 | 1 | 9 |
| 41 | 10 | 0 | 10 |
| 42 | 9 | 2 | 7 |
| 43 | 10 | 2 | 8 |
| 44 | 9 | 0 | 9 |
| 45 | 9 | 5 | 4 |
| 46 | 10 | 2 | 8 |
| 47 | 10 | 2 | 8 |
| 48 | 10 | 6 | 4 |
| 49 | 10 | 3 | 7 |
| 50 | 10 | 3 | 7 |
| 51 | 10 | 1 | 9 |
| 52 | 10 | 0 | 10 |
| Mean | 9.5 | 1.8 | 7.7 |
| SD | 1.0 | 1.8 | 2.0 |
| MIN | 5 | 0 | 4 |
| MAX | 11 | 6 | 11 |

| subject 3 | | | |
| --- | --- | --- | --- |
| cortical spot | number of  effective stimulations | number of  “leftward” answers | number of  “rightward” answers |
| 1 | 10 | 2 | 8 |
| 2 | 9 | 1 | 8 |
| 3 | 9 | 1 | 8 |
| 4 | 8 | 2 | 6 |
| 5 | 8 | 1 | 7 |
| 6 | 9 | 3 | 6 |
| 7 | 8 | 7 | 1 |
| 8 | 9 | 6 | 3 |
| 9 | 9 | 2 | 7 |
| 10 | 9 | 6 | 3 |
| 11 | 9 | 4 | 5 |
| 12 | 10 | 8 | 2 |
| 13 | 9 | 3 | 6 |
| 14 | 9 | 4 | 5 |
| 15 | 7 | 4 | 3 |
| 16 | 9 | 7 | 2 |
| 17 | 11 | 10 | 1 |
| 18 | 8 | 2 | 6 |
| 19 | 10 | 5 | 5 |
| 20 | 10 | 7 | 3 |
| 21 | 10 | 6 | 4 |
| 22 | 10 | 4 | 6 |
| 23 | 10 | 6 | 4 |
| 24 | 5 | 2 | 3 |
| 25 | 7 | 3 | 4 |
| 26 | 11 | 4 | 7 |
| 27 | 9 | 4 | 5 |
| 28 | 9 | 6 | 3 |
| 29 | 9 | 6 | 3 |
| 30 | 9 | 1 | 8 |
| 31 | 6 | 2 | 4 |
| 32 | 9 | 4 | 5 |
| 33 | 10 | 5 | 5 |
| 34 | 10 | 3 | 7 |
| 35 | 9 | 4 | 5 |
| 36 | 10 | 1 | 9 |
| 37 | 9 | 4 | 5 |
| 38 | 9 | 3 | 6 |
| 39 | 10 | 7 | 3 |
| 40 | 10 | 2 | 8 |
| 41 | 10 | 3 | 7 |
| 42 | 8 | 5 | 3 |
| 43 | 9 | 4 | 5 |
| 44 | 10 | 6 | 4 |
| 45 | 9 | 6 | 3 |
| 46 | 9 | 6 | 3 |
| 47 | 9 | 2 | 7 |
| 48 | 8 | 3 | 5 |
| 49 | 10 | 6 | 4 |
| 50 | 10 | 4 | 6 |
| 51 | 10 | 4 | 6 |
| 52 | 10 | 2 | 8 |
| Mean | 9.1 | 4.1 | 5.0 |
| SD | 1.1 | 2.1 | 2.0 |
| MIN | 5 | 1 | 1 |
| MAX | 11 | 10 | 9 |

| subject 4 | | | |
| --- | --- | --- | --- |
| cortical spot | number of  effective stimulations | number of  “leftward” answers | number of  “rightward” answers |
| 1 | 10 | 10 | 0 |
| 2 | 10 | 10 | 0 |
| 3 | 10 | 10 | 0 |
| 4 | 10 | 10 | 0 |
| 5 | 10 | 10 | 0 |
| 6 | 9 | 9 | 0 |
| 7 | 10 | 10 | 0 |
| 8 | 10 | 9 | 1 |
| 9 | 9 | 9 | 0 |
| 10 | 10 | 10 | 0 |
| 11 | 9 | 9 | 0 |
| 12 | 10 | 10 | 0 |
| 13 | 10 | 10 | 0 |
| 14 | 10 | 10 | 0 |
| 15 | 10 | 10 | 0 |
| 16 | 9 | 9 | 0 |
| 17 | 9 | 9 | 0 |
| 18 | 10 | 10 | 0 |
| 19 | 10 | 10 | 0 |
| 20 | 10 | 10 | 0 |
| 21 | 10 | 10 | 0 |
| 22 | 10 | 10 | 0 |
| 23 | 9 | 9 | 0 |
| 24 | 9 | 9 | 0 |
| 25 | 9 | 9 | 0 |
| 26 | 9 | 9 | 0 |
| 27 | 10 | 10 | 0 |
| 28 | 10 | 10 | 0 |
| 29 | 10 | 10 | 0 |
| 30 | 8 | 8 | 0 |
| 31 | 9 | 8 | 1 |
| 32 | 9 | 9 | 0 |
| 33 | 9 | 8 | 1 |
| 34 | 9 | 9 | 0 |
| 35 | 10 | 9 | 1 |
| 36 | 7 | 7 | 0 |
| 37 | 10 | 10 | 0 |
| 38 | 10 | 10 | 0 |
| 39 | 11 | 11 | 0 |
| 40 | 12 | 12 | 0 |
| 41 | 10 | 10 | 0 |
| 42 | 10 | 10 | 0 |
| 43 | 9 | 9 | 0 |
| 44 | 8 | 8 | 0 |
| 45 | 11 | 8 | 3 |
| 46 | 8 | 8 | 0 |
| 47 | 4 | 4 | 0 |
| 48 | 9 | 9 | 0 |
| 49 | 10 | 10 | 0 |
| 50 | 10 | 10 | 0 |
| 51 | 9 | 9 | 0 |
| 52 | 9 | 9 | 0 |
| Mean | 9.5 | 9.3 | 0.1 |
| SD | 1.1 | 1.1 | 0.5 |
| MIN | 4 | 4 | 0 |
| MAX | 12 | 12 | 3 |

| subject 5 | | | |
| --- | --- | --- | --- |
| cortical spot | number of  effective stimulations | number of  “leftward” answers | number of  “rightward” answers |
| 1 | 10 | 10 | 0 |
| 2 | 10 | 10 | 0 |
| 3 | 9 | 9 | 0 |
| 4 | 10 | 10 | 0 |
| 5 | 9 | 9 | 0 |
| 6 | 10 | 10 | 0 |
| 7 | 10 | 10 | 0 |
| 8 | 9 | 9 | 0 |
| 9 | 10 | 10 | 0 |
| 10 | 8 | 8 | 0 |
| 11 | 9 | 9 | 0 |
| 12 | 10 | 10 | 0 |
| 13 | 10 | 10 | 0 |
| 14 | 10 | 10 | 0 |
| 15 | 9 | 9 | 0 |
| 16 | 9 | 9 | 0 |
| 17 | 10 | 8 | 2 |
| 18 | 10 | 9 | 1 |
| 19 | 9 | 9 | 0 |
| 20 | 9 | 8 | 1 |
| 21 | 9 | 9 | 0 |
| 22 | 10 | 10 | 0 |
| 23 | 10 | 10 | 0 |
| 24 | 9 | 9 | 0 |
| 25 | 10 | 10 | 0 |
| 26 | 10 | 10 | 0 |
| 27 | 10 | 10 | 0 |
| 28 | 9 | 9 | 0 |
| 29 | 10 | 10 | 0 |
| 30 | 10 | 10 | 0 |
| 31 | 10 | 10 | 0 |
| 32 | 10 | 10 | 0 |
| 33 | 8 | 8 | 0 |
| 34 | 10 | 10 | 0 |
| 35 | 8 | 8 | 0 |
| 36 | 9 | 9 | 0 |
| 37 | 9 | 9 | 0 |
| 38 | 10 | 10 | 0 |
| 39 | 10 | 9 | 1 |
| 40 | 10 | 9 | 1 |
| 41 | 10 | 8 | 2 |
| 42 | 10 | 8 | 2 |
| 43 | 8 | 8 | 0 |
| 44 | 9 | 9 | 0 |
| 45 | 9 | 9 | 0 |
| 46 | 9 | 9 | 0 |
| 47 | 6 | 6 | 0 |
| 48 | 10 | 10 | 0 |
| 49 | 10 | 10 | 0 |
| 50 | 10 | 10 | 0 |
| 51 | 8 | 8 | 0 |
| 52 | 10 | 10 | 0 |
| Mean | 9.4 | 9.2 | 0.2 |
| SD | 0.8 | 0.9 | 0.5 |
| MIN | 6 | 6 | 0 |
| MAX | 10 | 10 | 2 |

| subject 6 | | | |
| --- | --- | --- | --- |
| cortical spot | number of  effective stimulations | number of  “leftward” answers | number of  “rightward” answers |
| 1 | 10 | 8 | 2 |
| 2 | 10 | 9 | 1 |
| 3 | 10 | 10 | 0 |
| 4 | 10 | 10 | 0 |
| 5 | 9 | 9 | 0 |
| 6 | 10 | 9 | 1 |
| 7 | 10 | 9 | 1 |
| 8 | 10 | 8 | 2 |
| 9 | 10 | 7 | 3 |
| 10 | 10 | 3 | 7 |
| 11 | 10 | 6 | 4 |
| 12 | 10 | 8 | 2 |
| 13 | 10 | 3 | 7 |
| 14 | 10 | 5 | 5 |
| 15 | 9 | 6 | 3 |
| 16 | 10 | 8 | 2 |
| 17 | 10 | 5 | 5 |
| 18 | 10 | 6 | 4 |
| 19 | 10 | 7 | 3 |
| 20 | 9 | 4 | 5 |
| 21 | 10 | 5 | 5 |
| 22 | 10 | 5 | 5 |
| 23 | 10 | 4 | 6 |
| 24 | 10 | 5 | 5 |
| 25 | 10 | 5 | 5 |
| 26 | 10 | 5 | 5 |
| 27 | 10 | 5 | 5 |
| 28 | 10 | 5 | 5 |
| 29 | 10 | 5 | 5 |
| 30 | 10 | 5 | 5 |
| 31 | 10 | 5 | 5 |
| 32 | 10 | 5 | 5 |
| 33 | 10 | 5 | 5 |
| 34 | 10 | 4 | 6 |
| 35 | 10 | 5 | 5 |
| 36 | 10 | 5 | 5 |
| 37 | 10 | 10 | 0 |
| 38 | 9 | 9 | 0 |
| 39 | 10 | 5 | 5 |
| 40 | 10 | 10 | 0 |
| 41 | 10 | 9 | 1 |
| 42 | 10 | 9 | 1 |
| 43 | 9 | 0 | 9 |
| 44 | 10 | 5 | 5 |
| 45 | 10 | 6 | 4 |
| 46 | 10 | 1 | 9 |
| 47 | 10 | 1 | 9 |
| 48 | 10 | 7 | 3 |
| 49 | 10 | 10 | 0 |
| 50 | 10 | 5 | 5 |
| 51 | 10 | 10 | 0 |
| 52 | 10 | 9 | 1 |
| Mean | 9.9 | 6.2 | 3.7 |
| SD | 0.3 | 2.5 | 2.5 |
| MIN | 9 | 0 | 0 |
| MAX | 10 | 10 | 9 |

| subject 7 | | | |
| --- | --- | --- | --- |
| cortical spot | number of  effective stimulations | number of  “leftward” answers | number of  “rightward” answers |
| 1 | 10 | 4 | 6 |
| 2 | 10 | 6 | 4 |
| 3 | 10 | 6 | 4 |
| 4 | 10 | 1 | 9 |
| 5 | 8 | 3 | 5 |
| 6 | 10 | 6 | 4 |
| 7 | 10 | 5 | 5 |
| 8 | 10 | 6 | 4 |
| 9 | 9 | 1 | 8 |
| 10 | 10 | 5 | 5 |
| 11 | 10 | 3 | 7 |
| 12 | 10 | 5 | 5 |
| 13 | 10 | 4 | 6 |
| 14 | 5 | 2 | 3 |
| 15 | 10 | 6 | 4 |
| 16 | 10 | 4 | 6 |
| 17 | 10 | 7 | 3 |
| 18 | 10 | 2 | 8 |
| 19 | 10 | 5 | 5 |
| 20 | 10 | 6 | 4 |
| 21 | 10 | 6 | 4 |
| 22 | 10 | 1 | 9 |
| 23 | 10 | 5 | 5 |
| 24 | 10 | 6 | 4 |
| 25 | 10 | 6 | 4 |
| 26 | 10 | 6 | 4 |
| 27 | 10 | 2 | 8 |
| 28 | 10 | 8 | 2 |
| 29 | 10 | 5 | 5 |
| 30 | 10 | 0 | 10 |
| 31 | 10 | 4 | 6 |
| 32 | 10 | 7 | 3 |
| 33 | 10 | 2 | 8 |
| 34 | 10 | 1 | 9 |
| 35 | 9 | 7 | 2 |
| 36 | 10 | 4 | 6 |
| 37 | 10 | 1 | 9 |
| 38 | 10 | 4 | 6 |
| 39 | 10 | 4 | 6 |
| 40 | 10 | 6 | 4 |
| 41 | 10 | 3 | 7 |
| 42 | 10 | 6 | 4 |
| 43 | 10 | 3 | 7 |
| 44 | 10 | 2 | 8 |
| 45 | 10 | 9 | 1 |
| 46 | 10 | 4 | 6 |
| 47 | 9 | 5 | 4 |
| 48 | 10 | 3 | 7 |
| 49 | 10 | 4 | 6 |
| 50 | 10 | 5 | 5 |
| 51 | 10 | 5 | 5 |
| 52 | 10 | 7 | 3 |
| Mean | 9.8 | 4.4 | 5.4 |
| SD | 0.8 | 2.0 | 2.0 |
| MIN | 5 | 0 | 1 |
| MAX | 10 | 9 | 10 |

| subject 8 | | | |
| --- | --- | --- | --- |
| cortical spot | number of  effective stimulations | number of  “leftward” answers | number of  “rightward” answers |
| 1 | 10 | 10 | 0 |
| 2 | 10 | 10 | 0 |
| 3 | 10 | 10 | 0 |
| 4 | 10 | 9 | 1 |
| 5 | 10 | 10 | 0 |
| 6 | 10 | 10 | 0 |
| 7 | 10 | 8 | 2 |
| 8 | 10 | 10 | 0 |
| 9 | 10 | 10 | 0 |
| 10 | 10 | 9 | 1 |
| 11 | 10 | 10 | 0 |
| 12 | 10 | 10 | 0 |
| 13 | 10 | 10 | 0 |
| 14 | 10 | 10 | 0 |
| 15 | 10 | 9 | 1 |
| 16 | 10 | 10 | 0 |
| 17 | 10 | 10 | 0 |
| 18 | 10 | 10 | 0 |
| 19 | 10 | 10 | 0 |
| 20 | 10 | 10 | 0 |
| 21 | 10 | 10 | 0 |
| 22 | 10 | 10 | 0 |
| 23 | 10 | 9 | 1 |
| 24 | 10 | 9 | 1 |
| 25 | 10 | 7 | 3 |
| 26 | 10 | 10 | 0 |
| 27 | 10 | 10 | 0 |
| 28 | 10 | 10 | 0 |
| 29 | 10 | 10 | 0 |
| 30 | 10 | 10 | 0 |
| 31 | 10 | 10 | 0 |
| 32 | 10 | 10 | 0 |
| 33 | 10 | 9 | 1 |
| 34 | 10 | 10 | 0 |
| 35 | 10 | 10 | 0 |
| 36 | 10 | 7 | 3 |
| 37 | 10 | 10 | 0 |
| 38 | 10 | 8 | 2 |
| 39 | 10 | 7 | 3 |
| 40 | 10 | 10 | 0 |
| 41 | 10 | 10 | 0 |
| 42 | 10 | 10 | 0 |
| 43 | 9 | 6 | 3 |
| 44 | 10 | 10 | 0 |
| 45 | 10 | 10 | 0 |
| 46 | 10 | 8 | 2 |
| 47 | 10 | 9 | 1 |
| 48 | 10 | 10 | 0 |
| 49 | 10 | 9 | 1 |
| 50 | 10 | 9 | 1 |
| 51 | 10 | 10 | 0 |
| 52 | 10 | 9 | 1 |
| Mean | 10.0 | 9.4 | 0.5 |
| SD | 0.1 | 1.0 | 0.9 |
| MIN | 9 | 6 | 0 |
| MAX | 10 | 10 | 3 |

| subject 9 | | | |
| --- | --- | --- | --- |
| cortical spot | number of  effective stimulations | number of  “leftward” answers | number of  “rightward” answers |
| 1 | 10 | 7 | 3 |
| 2 | 10 | 9 | 1 |
| 3 | 10 | 8 | 2 |
| 4 | 10 | 10 | 0 |
| 5 | 10 | 9 | 1 |
| 6 | 10 | 9 | 1 |
| 7 | 10 | 10 | 0 |
| 8 | 10 | 8 | 2 |
| 9 | 10 | 10 | 0 |
| 10 | 10 | 7 | 3 |
| 11 | 10 | 8 | 2 |
| 12 | 10 | 9 | 1 |
| 13 | 10 | 10 | 0 |
| 14 | 10 | 9 | 1 |
| 15 | 10 | 8 | 2 |
| 16 | 10 | 10 | 0 |
| 17 | 10 | 8 | 2 |
| 18 | 10 | 8 | 2 |
| 19 | 10 | 9 | 1 |
| 20 | 10 | 9 | 1 |
| 21 | 10 | 7 | 3 |
| 22 | 10 | 7 | 3 |
| 23 | 10 | 6 | 4 |
| 24 | 10 | 9 | 1 |
| 25 | 10 | 8 | 2 |
| 26 | 10 | 8 | 2 |
| 27 | 10 | 9 | 1 |
| 28 | 10 | 9 | 1 |
| 29 | 10 | 7 | 3 |
| 30 | 10 | 8 | 2 |
| 31 | 9 | 8 | 1 |
| 32 | 10 | 6 | 4 |
| 33 | 10 | 7 | 3 |
| 34 | 9 | 8 | 1 |
| 35 | 9 | 9 | 0 |
| 36 | 10 | 8 | 2 |
| 37 | 10 | 10 | 0 |
| 38 | 10 | 10 | 0 |
| 39 | 10 | 10 | 0 |
| 40 | 10 | 9 | 1 |
| 41 | 10 | 6 | 4 |
| 42 | 10 | 7 | 3 |
| 43 | 10 | 10 | 0 |
| 44 | 10 | 9 | 1 |
| 45 | 10 | 10 | 0 |
| 46 | 10 | 10 | 0 |
| 47 | 10 | 10 | 0 |
| 48 | 10 | 7 | 3 |
| 49 | 10 | 10 | 0 |
| 50 | 10 | 10 | 0 |
| 51 | 10 | 10 | 0 |
| 52 | 10 | 7 | 3 |
| Mean | 9.9 | 8.5 | 1.4 |
| SD | 0.2 | 1.2 | 1.3 |
| MIN | 9 | 6 | 0 |
| MAX | 10 | 10 | 4 |

| subject 10 | | | |
| --- | --- | --- | --- |
| cortical spot | number of  effective stimulations | number of  “leftward” answers | number of  “rightward” answers |
| 1 | 10 | 7 | 3 |
| 2 | 9 | 6 | 3 |
| 3 | 10 | 9 | 1 |
| 4 | 9 | 9 | 0 |
| 5 | 10 | 9 | 1 |
| 6 | 10 | 8 | 2 |
| 7 | 10 | 9 | 1 |
| 8 | 10 | 10 | 0 |
| 9 | 10 | 9 | 1 |
| 10 | 10 | 10 | 0 |
| 11 | 10 | 10 | 0 |
| 12 | 10 | 9 | 1 |
| 13 | 10 | 10 | 0 |
| 14 | 10 | 10 | 0 |
| 15 | 10 | 10 | 0 |
| 16 | 9 | 9 | 0 |
| 17 | 10 | 9 | 1 |
| 18 | 10 | 10 | 0 |
| 19 | 10 | 9 | 1 |
| 20 | 10 | 10 | 0 |
| 21 | 10 | 9 | 1 |
| 22 | 10 | 10 | 0 |
| 23 | 10 | 9 | 1 |
| 24 | 10 | 10 | 0 |
| 25 | 10 | 10 | 0 |
| 26 | 10 | 9 | 1 |
| 27 | 9 | 9 | 0 |
| 28 | 10 | 9 | 1 |
| 29 | 10 | 10 | 0 |
| 30 | 10 | 9 | 1 |
| 31 | 10 | 9 | 1 |
| 32 | 10 | 10 | 0 |
| 33 | 10 | 10 | 0 |
| 34 | 10 | 9 | 1 |
| 35 | 10 | 10 | 0 |
| 36 | 10 | 9 | 1 |
| 37 | 10 | 9 | 1 |
| 38 | 10 | 10 | 0 |
| 39 | 10 | 9 | 1 |
| 40 | 10 | 9 | 1 |
| 41 | 9 | 9 | 0 |
| 42 | 10 | 8 | 2 |
| 43 | 10 | 8 | 2 |
| 44 | 10 | 9 | 1 |
| 45 | 10 | 8 | 2 |
| 46 | 8 | 5 | 3 |
| 47 | 10 | 10 | 0 |
| 48 | 10 | 9 | 1 |
| 49 | 10 | 7 | 3 |
| 50 | 10 | 9 | 1 |
| 51 | 10 | 10 | 0 |
| 52 | 8 | 6 | 2 |
| Mean | 9.8 | 9.0 | 0.8 |
| SD | 0.5 | 1.1 | 0.9 |
| MIN | 8 | 5 | 0 |
| MAX | 10 | 10 | 3 |
